# Treatment of anti-GAD65 antibody-positive epilepsy and cerebellar ataxia with efgartigimod: a case report and literature review

**DOI:** 10.3389/fimmu.2026.1849117

**Published:** 2026-08-04

**Authors:** Lulu Xie, Junqin Zuo, Le Luo, Duo Zhang, Zhuang Ma, Zihan Wang, Chunying Deng

**Affiliations:** 1 Tianjin Academy of Traditional Chinese Medicine, Tianjin, China; 2 First Department of Neurology, North China University of Science and Technology Affiliated Hospital, Tangshan, China; 3 Department of Neurology II, Tianjin Academy of Traditional Chinese Medicine Affiliated Hospital, Tianjin, China

**Keywords:** case report, cerebellar ataxia, efgartigimod, epilepsy, GAD65

## Abstract

**Background:**

Anti-glutamic acid decarboxylase 65 (GAD65) antibody-associated neurological syndrome is a rare autoimmune disorder with heterogeneous clinical manifestations, including epilepsy and cerebellar ataxia. Standard treatment strategies remain limited. This report aimed to describe a rare case presenting with combined epilepsy and cerebellar ataxia, and to evaluate the efficacy of efgartigimod followed by mycophenolate mofetil.

**Methods:**

We present a 62-year-old female with slowly progressive recurrent epileptic seizures and cerebellar ataxia. Clinical, neurological, neuroimaging (PET/CT, MRI), and laboratory examinations (serum and cerebrospinal fluid GAD65 antibodies) were analyzed. The patient was treated with efgartigimod (10 mg/kg weekly for 3 doses) plus maintenance mycophenolate mofetil. Clinical outcomes and follow-up data were assessed.

**Results:**

Neurological examination showed left-sided dysmetria, dysdiadochokinesia, and a positive Romberg sign. Brain PET/CT revealed hypometabolism in the bilateral temporal lobes, right hippocampus, and left frontoparietal regions, accompanied by mild right hippocampal atrophy. Serum and cerebrospinal fluid (CSF) examinations were positive for GAD65 antibodies at titers of 1:1000 and 1:100, respectively, confirming the diagnosis of GAD65 antibody-associated neurological syndrome. After treatment, seizures completely resolved, gait ataxia improved significantly, and daily activities were fully restored at 1-month follow-up. Antiseizure medications were gradually tapered. No clinical relapse occurred during the 1-year follow-up period.

**Conclusions:**

This case demonstrates that anti-GAD65 antibody syndrome can present as concurrent refractory epilepsy and cerebellar ataxia. The sequential regimen of efgartigimod induction combined with mycophenolate mofetil maintenance appears effective and safe. This therapeutic strategy may provide a valuable reference for the clinical management of such rare autoimmune neurological disorders.

## Introduction

Glutamic acid decarboxylase-65 (GAD65) is a key enzyme responsible for the biosynthesis of gamma-aminobutyric acid, the primary inhibitory neurotransmitter in the central nervous system (1). GAD65 antibodies serve as a biomarker for autoimmune disorders, which are characterized by immune-mediated attack on the nervous and endocrine systems by anti-GAD65 antibodies. Anti-GAD65 antibody-related autoimmune disorders can be categorized into neurological and non-neurological manifestations. Neurological phenotypes mainly include stiff-person syndrome (SPS), autoimmune epilepsy, limbic encephalitis (LE), and cerebellar ataxia (CA), whereas non-neurological manifestations predominantly consist of diabetes mellitus type 1 (T1DM) (2). Space-occupying lesions are present in only a minority of cases. Given the low incidence and heterogeneous clinical spectrum of anti-GAD65 antibody-associated disorders, standardized treatment protocols have not yet been established, and current management largely relies on combined immunotherapy. Adjunctive measures include seizure control, antineoplastic therapy, and other symptomatic interventions, which should be individually optimized according to patient-specific clinical features.

Based on previous reports, immunotherapy is the established mainstay for anti-GAD65-related diseases (3). Early and intensive immunotherapy is correlated with a more favorable prognosis. Immunotherapeutic strategies are generally classified into first-line, second-line, long-term maintenance, escalation, and additive regimens. It is broadly recommended that all patients with newly diagnosed encephalitis receive first-line immunotherapy, to which more than half of affected individuals exhibit a satisfactory response (4). However, conventional immunotherapy frequently demonstrates limited efficacy in reducing seizure frequency and ameliorating cognitive impairment. As illustrated in the current case, second-line immunotherapy should be promptly initiated in patients who fail to achieve substantial clinical improvement following adequate trials of two or more first-line agents (5). Given the intracellular localization of the GAD65 antigen, systemic immunotherapies generally yield poor clinical effects in most patients, and no unified standard immunotherapy protocols are available for these diseases at present.

This report describes the clinical features and therapeutic management of a patient with anti-GAD65 antibody-associated epilepsy and cerebellar ataxia, with the aim of improving clinical recognition and providing novel references for treatment strategies.

## Case presentation

A 62-year-old female was admitted to our hospital on October 8, 2024, with a chief complaint of episodic loss of consciousness and focal clonic activity involving the limbs for 20 years. Three months before admission, the patient developed progressive cerebellar ataxia, presenting as persistent unsteady gait with lateral swaying that required assistive devices for ambulation. This was accompanied by dizziness, vertigo, nausea and vomiting, leading to multiple falls during this period.

The patient’s medical history and treatment process are described as follows:

Her initial neurological manifestations onset in 2004, presenting as brief staring spells (duration: tens of seconds) accompanied by drooling from the left oral commissure. She visited a local hospital at that time without a clear diagnosis. Oral carbamazepine of unknown dosage was prescribed but failed to relieve her symptoms, with seizures occurring 50–60 times per year. The clinical course was progressively deteriorating, with subsequent evolution into bilateral tonic-clonic seizures, characterized by generalised seizures, frothy saliva, cyanosis, tongue biting and urinary incontinence. In the postictal phase, the patient suffered from left temporal and occipital headache, whose frequency and intensity were aggravated by emotional stress and sleep disturbance.

**Figure 1:**
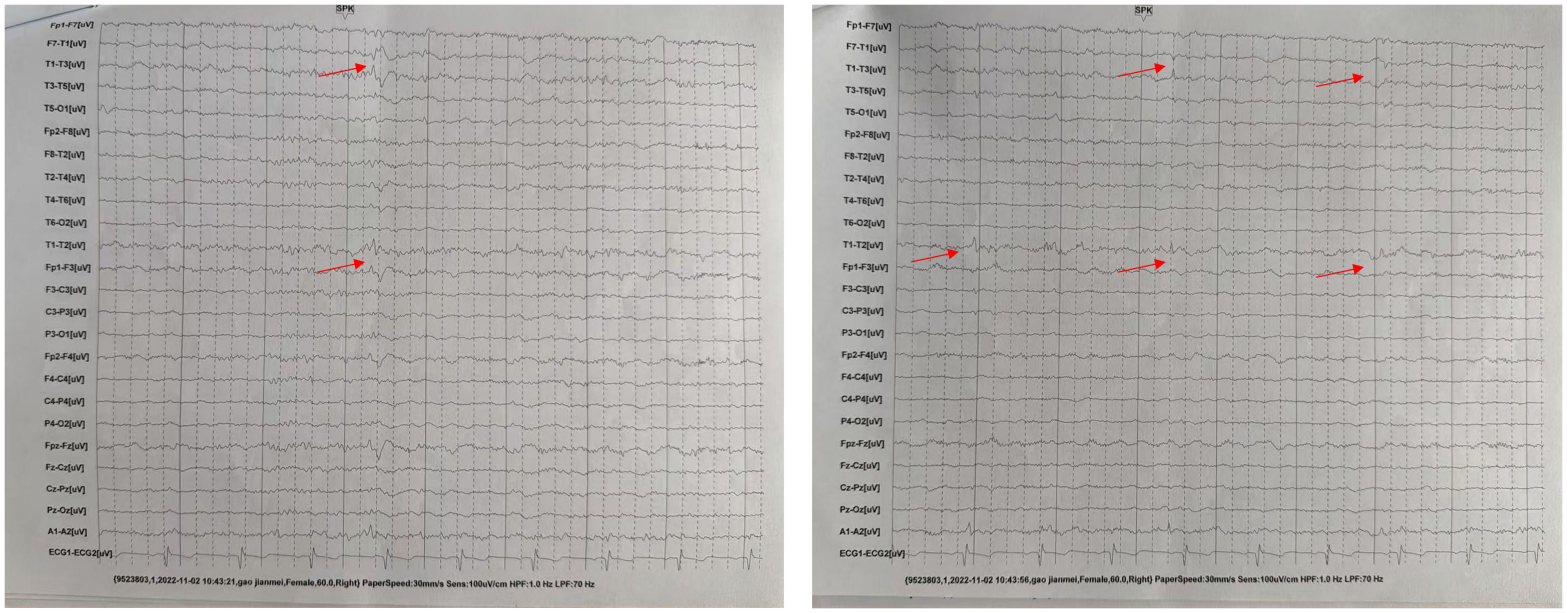
Brain EEG.

In 2015, brain MRI and 24-hour EEG (**Figure 1**) were completed, and epilepsy was diagnosed. She was treated sequentially with multiple antiseizure medications: oxcarbazepine 450 mg twice daily, levetiracetam 1 g twice daily, valproate 500 mg twice daily and zonisamide 150 mg twice daily, none of which yielded effective seizure control.

In 2022, the patient’s antiseizure medication regimen was optimized at Beijing Tiantan Hospital. She was prescribed oral lacosamide 300 mg twice daily, oral levetiracetam 0.5 g twice daily, and oral perampanel 4 mg once nightly. Notably, the patient frequently adjusted her medication dosage without medical guidance. Despite this combination therapy, episodes of blank staring and teeth grinding persisted, occurring 3–4 times monthly.

On October 12, 2023, brain PET/CT imaging revealed hypometabolism suggestive of focal lesions in the anterior right temporal lobe, reduced metabolic activity in the right hippocampus, mild volumetric atrophy of the right hippocampus and right temporal lobe, hypometabolism within the left temporal lobe, and multifocal FDG hypometabolism in the left frontal and parietal lobes with the most prominent changes in the left frontal lobe. Positive anti-GAD65 antibodies were detected at a titer of 1:320, leading to a final diagnosis of autoimmune encephalitis. Intravenous human immunoglobulin was administered during hospitalization, which partially reduced seizure frequency. After discharge, the patient adhered to a stable regimen of oral lacosamide 300 mg twice daily, oral levetiracetam 0.5 g twice daily, and oral perampanel 4 mg once nightly. The patient had a 12-year history of type 2 diabetes mellitus. Her regular hypoglycemic regimen consisted of oral acarbose 50 mg three times daily, oral metformin 0.5 g three times daily, and subcutaneous glargine insulin 13 units once nightly, yet her glycemic control remained suboptimal. Despite sequential therapeutic trials of multiple antiseizure medications, including carbamazepine, oxcarbazepine, levetiracetam, sodium valproate, zonisamide, lacosamide and perampanel, satisfactory seizure control was not achieved.

On admission, vital signs were stable: temperature 36.2 °C, pulse 80 beats/min, respiratory rate 20 breaths/min, and blood pressure 130/77 mmHg. General physical examination showed no abnormal findings in the cardiovascular, respiratory, or abdominal systems. Neurological examination revealed clear consciousness, fluent speech, intact extraocular movements without nystagmus, and bilaterally normal-sized pupils with preserved light reaction. Facial symmetry was intact, and the tongue protruded midline. Muscle strength was graded 5 throughout all extremities, with normal muscle tone. Deep tendon reflexes were absent at the knees and ankles bilaterally; plantar responses were flexor (negative Babinski and Chaddock signs). Sensory examination was unremarkable. Cerebellar evaluation demonstrated left-sided dysmetria on finger-to-nose and heel-knee-shin testing, along with ipsilateral dysdiadochokinesia. The Romberg sign was positive both with eyes open and closed. No meningeal irritation signs were observed. The Scale for the Assessment and Rating of Ataxia (SARA) score on admission was 17.

**Figure 2:**
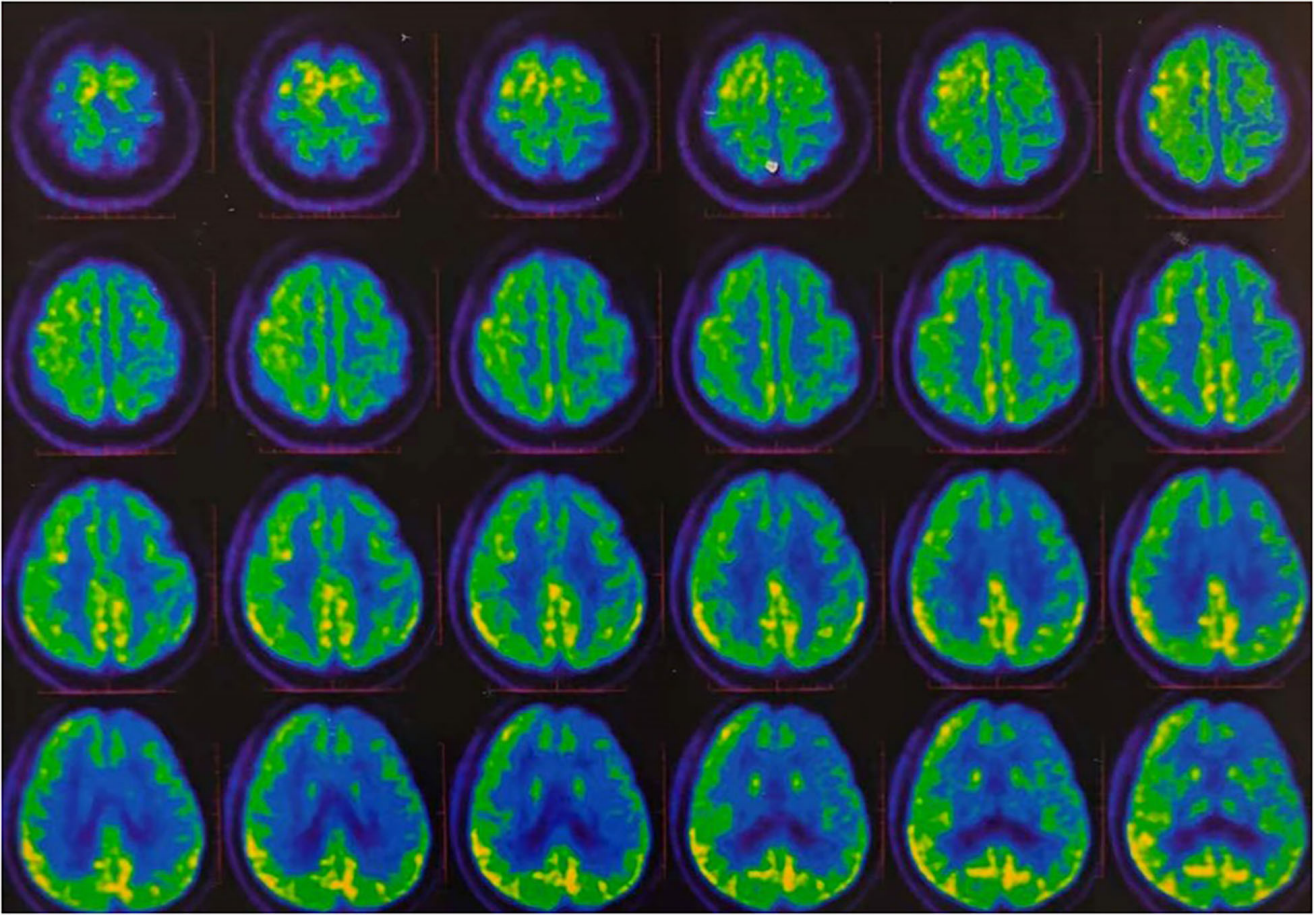
Brain PET/CT.

**Figure 3:**
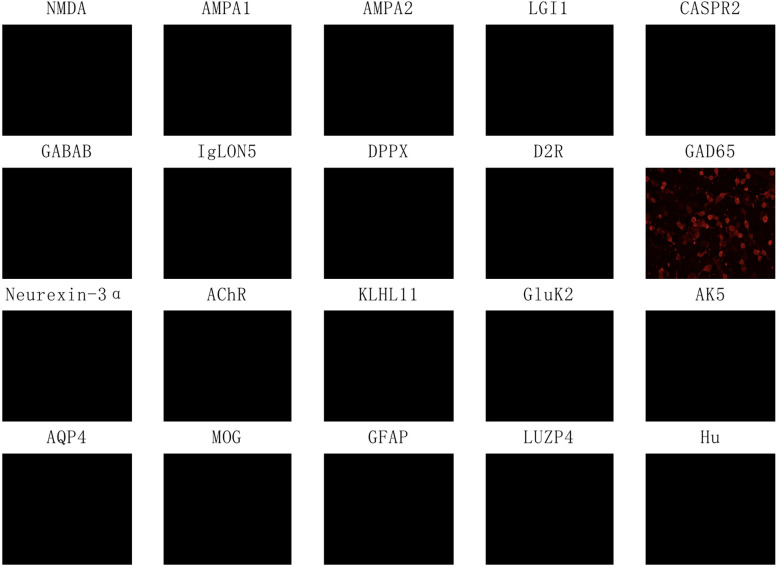
Fluorescence image results of GAD65 antibody.

Brain PET/CT performed on October 12, 2023, demonstrated hypometabolism involving the anterior right temporal lobe, right hippocampus, and left temporal lobe, accompanied by mild volumetric reduction of the right hippocampus (**Figure 2**). Cranial MRI conducted on October 12, 2024, revealed multiple chronic ischemic infarcts and diffuse cerebral atrophy. Serological and CSF analyses obtained on October 9, 2024, confirmed positivity for glutamic acid decarboxylase 65 (GAD65) antibodies, with serum (**Table 1**) and CSF (**Table 2**) titers of 1:1000 and 1:100 respectively. The immunofluorescence image of GAD65 is shown in **Figure 3**. CSF protein was mildly elevated at 0.44 g/L. Combining the clinical presentations of refractory epilepsy and progressive cerebellar ataxia, characteristic neuroimaging alterations, and serologically verified GAD65 antibody positivity, a definitive diagnosis of GAD65 antibody-associated neurological syndrome was rendered.

**Table 1:** Results of GAD65 antibody in serum.

Item	Testing method	Result	Reference range
NMDAR antibody	CBA method	Negative (-)	Negative (-)
AMPA1 antibody	CBA method	Negative (-)	Negative (-)
AMPA2 antibody	CBA method	Negative (-)	Negative (-)
LGI1 antibody	CBA method	Negative (-)	Negative (-)
CASPR2 antibody	CBA method	Negative (-)	Negative (-)
GlyR1 antibody	CBA method	Negative (-)	Negative (-)
GABAA antibody	CBA method	Negative (-)	Negative (-)
GABAB antibody	CBA method	Negative (-)	Negative (-)
IgLON5 antibody	CBA method	Negative (-)	Negative (-)
DPPX antibody	CBA method	Negative (-)	Negative (-)
DRD2 antibody	CBA method	Negative (-)	Negative (-)
GAD65 antibody	CBA method	**Positive (1:1000+++)**	Negative (-)

Bold values denote positive test results of GAD65 Antibody in serum.

**Table 2:** Results of GAD65 antibody in CSF.

Item	Testing method	Result	Reference range
Titin antibody	CBA method	Negative (-)	Negative (-)
Recoverin antibody	CBA method	Negative (-)	Negative (-)
PKCγ antibody	CBA method	Negative (-)	Negative (-)
NMDAR antibody	CBA method	Negative (-)	Negative (-)
AMPA1 antibody	CBA method	Negative (-)	Negative (-)
AMPA2 antibody	CBA method	Negative (-)	Negative (-)
LGI 1 antibody	CBA method	Negative (-)	Negative (-)
CASPR2 antibody	CBA method	Negative (-)	Negative (-)
GlyR1 antibody	CBA method	Negative (-)	Negative (-)
GABAA antibody	CBA method	Negative (-)	Negative (-)
GABAB antibody	CBA method	Negative (-)	Negative (-)
GAD65 antibody	CBA method	**Positive (1:100+++)**	Negative (-)

Bold values denote positive test results of GAD65 Antibody in CSF.

Following admission, the patient received efgartigimod infusions at a dosage of 10 mg/kg weekly for a total of three doses. This therapeutic intervention led to a marked reduction in seizure frequency and significant improvement in cerebellar ataxia, as reflected by a decrease in the SARA score from 17 to 8. The patient subsequently regained independent ambulation. Upon discharge, maintenance immunosuppressive treatment with mycophenolate mofetil dispersible tablets (0.5g twice daily) was instituted, with regular surveillance of complete blood count and immunological parameters. At the one-month follow-up evaluation, the patient had fully resumed normal daily activities. At four months post-discharge (February 17, 2025), serum anti-GAD65 antibody titers remained positive at 1:1000, while IgG levels and peripheral B-cell proportions exhibited a decreasing trend. No clinical relapse was documented throughout the one-year follow-up period. Summary of clinical course, auxiliary examinations, treatment regimens and clinical response (**Table 3**).

**Table 3:** Summary of clinical course, auxiliary examinations, treatment regimens and clinical response.

Time point	Key clinical manifestations	Examinations	Treatment regimens	Clinical response
2004 (Initial onset, 20 years before admission)	Brief staring spells, drooling from left mouth; later developed bilateral tonic-clonic seizures (frothing, lip cyanosis, tongue biting, urinary incontinence); left temporal-occipital headache; seizure frequency: 50–60 times/year	Not tested	Carbamazepine (unknown dosage)	No seizure relief
2015	Persistent refractory seizures	Brain MRI + 24 h EEG: diagnosed epilepsy	Oxcarbazepine 450 mg twice daily; levetiracetam 1 g twice daily; valproate 500 mg twice daily; zonisamide 150 mg twice daily	Failed seizure control
2022	Frequent blank staring, teeth grinding; seizures 3–4 times/month	No repeat neuroimaging	Lacosamide 300 mg twice daily; levetiracetam 0.5 g twice daily; perampanel 4 mg once nightly (patient self-adjusted dosage irregularly)	Seizures persisted
12 Oct 2023	Seizures unchanged	FDG PET/CT: hypometabolism of anterior right temporal lobe, right hippocampus, left temporal lobe, left frontoparietal lobe; mild right hippocampal atrophySerum anti-GAD65 antibody 1:320	lacosamide 300 mg twice daily, oral levetiracetam 0.5 g twice daily, and oral perampanel 4 mg once;nightlyIntravenous human immunoglobulin	Partial reduction of seizure frequency
Admission (8 Oct 2024)	Refractory focal seizures; severe cerebellar ataxia, SARA score = 17; left dysmetria, dysdiadochokinesia, positive Romberg sign	Brain MRI: multiple chronic ischemic infarcts, diffuse cerebral atrophy Serum anti-GAD65 1:1000; CSF anti-GAD65 1:100; all other neuronal autoantibodies negative; CSF protein 0.44 g/L	Lacosamide 300 mg twice daily; levetiracetam 0.5 g twice daily; perampanel 4 mg once nightly Efgartigimod 10 mg/kg weekly × 3 doses	Marked improvement of seizures and ataxia; SARA decreased to 8; independent walking recovered
1-month follow-up (Nov 2024)	No seizure recurrence; normal daily activities	Not rechecked	Tapering ASMs gradually Continuous mycophenolate mofetil	Complete recovery of daily living ability
4-month follow-up (17 Feb 2025)	No seizures; stable gait function	Serum anti-GAD65 still positive 1:1000; IgG and peripheral B cell count declined	Low-dose maintenance ASMs Continuous mycophenolate mofetil	Sustained remission
1-year follow-up (Oct 2025)	No clinical relapse	No repeated antibody testing	Long-term low-dose ASMsContinuous mycophenolate mofetil	No recurrence of seizures

## Discussion

GAD65 is widely expressed throughout the nervous system. In patients with anti-GAD65 antibodies, brain MRI frequently shows temporal lobe and hippocampal involvement. The main clinical manifestations include epileptic seizures and cognitive impairment, while cerebellar ataxia is also a well-recognized phenotype. The disease usually follows a subacute or chronic progressive course, is mostly sporadic, and predominantly affects middle-aged to elderly women (6). Gait ataxia is the most common presentation, followed by limb ataxia, diplopia, and brainstem-related dysarthria (2). These symptoms can occur simultaneously or sequentially during disease progression (7). Our patient initially presented with seizures and subsequently developed cerebellar ataxia. The treatment strategy reported herein provides clinical reference for the management of anti-GAD65 antibody-positive patients complicated by both epilepsy and cerebellar ataxia.

Anti-GAD65 antibodies disrupt the conversion of glutamate to GABA via humoral immunity, leading to increased glutamate levels, enhanced neuronal excitability, and reduced seizure threshold (8, 9). Recent studies suggest anti-GAD65 antibodies may directly promote epileptogenesis (10, 11). In contrast, some research proposes cytotoxic T-cell-mediated neuronal injury as the primary mechanism, with anti-GAD65 antibodies acting only as an immunological marker rather than directly pathogenic (12).

In anti-GAD65–associated autoimmune epilepsy, temporal lobe epilepsy is the typical phenotype, often accompanied by chronic encephalopathy. Focal to bilateral tonic-clonic seizures and focal non-motor seizures are the most common seizure types (13). Multiple seizure types may develop over time, as seen in our patient. Cerebellar atrophy has been reported in some patients with anti-GAD65 associated ataxia (14), but was not obvious in our case. The definite relationship between anti-GAD65 antibodies and cerebellar atrophy, as well as its underlying mechanism, requires further large-scale studies.

Approximately half of patients with anti-GAD65 antibodies have concomitant systemic autoimmune diseases, mainly type 1 diabetes, followed by autoimmune thyroiditis (15, 16). The absence of systemic autoimmunity in our patient suggests a relatively favorable prognosis. Current treatment for anti-GAD65 related neurological syndromes combines immunotherapy, antiseizure medications. First-line immunotherapy includes high-dose intravenous methylprednisolone, intravenous immunoglobulin, and plasma exchange (17). Second-line options include rituximab and cyclophosphamide. However, first-line treatments carry side effects or high costs, and rituximab efficacy remains inconsistent in some reports (18). ASM monotherapy or even combination therapy often fails to achieve satisfactory seizure control in refractory cases, as observed in our patient. For patients with inadequate response to first- and second-line immunotherapy, long-term maintenance immunosuppression is recommended (11). Mycophenolate mofetil is a reasonable choice (19).

Efgartigimod, an FcRn antagonist that accelerates IgG degradation, has shown promise in anti-GAD-associated neurological autoimmunity. Previous cases reported improvement in myasthenia gravis and stiff-person syndrome after efgartigimod treatment, with reduced autoantibody titers (20). Efgartigimod can lower pathogenic IgG levels, reduce relapse risk, improve clinical outcomes, and avoid steroid-related toxicities (21). In our patient, three weekly infusions of efgartigimod led to rapid and marked improvement in seizures and ataxia, with no relapse during follow-up.

## Conclusions

This report presents a unique case of anti-GAD65 antibody-associated neurological disorder manifesting as coexisting epileptic seizures and cerebellar ataxia. The patient achieved substantial clinical improvement following sequential immunotherapy with efgartigimod and mycophenolate mofetil. The heterogeneous treatment responses reported in previous literature underscore the therapeutic challenges in managing anti-GAD65–related neurological autoimmunity, rendering our clinical experience highly valuable for clinical practice.

Increasing recognition of complex autoantibody profiles in neuroimmunological disorders demands refined therapeutic strategies. With growing clinical awareness, patients often present with multiple autoantibodies and overlapping clinical phenotypes. Therefore, developing optimized therapeutic regimens that maximize efficacy while minimizing adverse effects of conventional immunosuppressive agents has become an urgent priority for future research. Large-scale prospective studies are warranted to validate these therapeutic approaches and establish evidence-based clinical guidelines.

## Data Availability

The original contributions presented in the study are included in the article/Supplementary Material. Further inquiries can be directed to the corresponding author.
